# Phytochemical Profile of *Cymbopogon citratus* (DC.) Stapf Lemongrass Essential Oil from Northeastern Thailand and Its Antioxidant and Antimicrobial Attributes and Cytotoxic Effects on HT-29 Human Colorectal Adenocarcinoma Cells

**DOI:** 10.3390/foods13182928

**Published:** 2024-09-15

**Authors:** Vijitra Luang-In, Worachot Saengha, Thipphiya Karirat, Chadaporn Senakun, Sirithon Siriamornpun

**Affiliations:** 1 Natural Antioxidant Innovation Research Unit, Department of Biotechnology, Faculty of Technology, Mahasarakham University, Kantarawichai, Mahasarakham 44150, Thailand; vijitra.l@msu.ac.th (V.L.-I.); worachot207@gmail.com (W.S.); thipphiya.k@gmail.com (T.K.); 2 Walai Rukhavej Botanical Research Institute, Mahasarakham University, Kantarawichai, Mahasarakham 44150, Thailand; chadaporn.sen@gmail.com; 3 Research Unit of Thai Food Innovation (TFI), Mahasarakham University, Kantarawichai, Mahasarakham 44150, Thailand; 4 Department of Food Technology and Nutrition, Faculty of Technology, Mahasarakham University, Kantarawichai, Mahasarakham 44150, Thailand

**Keywords:** apoptosis, colon cancer, cytotoxicity, herbal plant extract, pathogens

## Abstract

Colorectal cancer is the third most prevalent cancer in Thailand, prompting the search for alternative or preventive treatments using natural constituents. In this study, the authors employed hydrodistillation to extract *Cymbopogon citratus* (DC.) Stapf (lemongrass) essential oil (LEO) from plants in northeastern Thailand and assessed its chemical profile, antioxidant, antimicrobial, and anticancer properties. The LEO displayed potent antioxidant activities in DPPH and hydroxyl scavenging assays with IC_50_ values of 2.58 ± 0.08 and 4.05 ± 0.12 mg/mL, respectively, and demonstrated antimicrobial activities against *Escherichia coli*, *Cutibacterium acnes*, *Streptococcus agalactiae*, and *Staphylococcus aureus* at 8–10 µg/mL. At 48 h, the 3-[4,5-dimethylthiazol-2-yl]-2,5 diphenyl tetrazolium bromide (MTT) assay showed the LEO exhibiting low cell viability (3%) at concentrations of 200–400 µg/mL, with an IC_50_ value of 82.46 ± 1.73 µg/mL, while in the clonogenic assay it exhibited a lower IC_50_ value of 23.11 ± 1.80 µg/mL. The GC-MS analysis identified citral (79.24%) consisting of 44.52% geranial and 34.72% neral, and β-myrcene (5.56%). The addition of LEO significantly influenced apoptotic genes (*Bcl-2*, *Bax*, *p21*, and *Caspase-3*) and proteins, as indicated by real-time polymerase chain reaction (RT-PCR) and Western blot studies. Results suggested that LEO initiated apoptosis through intrinsic pathways and demonstrated potential as a chemopreventive, antimicrobial, and antioxidant agent with substantial health advantages.

## 1. Introduction

Colorectal cancer (CRC) ranks third among Thailand’s most common cancers, constituting 11% of the cancer burden [1]. The prevalence of CRC in Thailand has consistently risen among males and females. Follicleronid, 5-fluorouracil, and oxaliplatin, or FOLFOX are the chemotherapy drugs most commonly used to treat CRC. Despite its short-term effectiveness, this medicine is not recommended for long-term use because of substantial gastrointestinal and neurotoxic adverse effects [2]. Natural compounds derived from medicinal plants with potential anticancer properties have recently gained attention.

Natural products have long been used for their therapeutic characteristics and diverse uses. However, they are not always appropriate as therapies or supplements due to the lack of scientific evidence and validation through experiments. Many modern anticancer drugs are derived from or chemically similar to plant substances [3,4]. Natural products are cheap and abundant, with increasing development as alternative and effective cancer treatments.

Lemongrass (*Cymbopogon citratus* (DC.) Stapf), a member of the Poaceae family, is a common ingredient in Asian cuisine and is also used as a medicinal herb [5]. Lemongrass essential oil (LEO) has a pleasant scent and is derived from this perennial plant that is extensively grown in tropical regions [6]. Shah et al. (2011) found that the chemical composition of LEO varied based on growth conditions, harvest timing, and plant material origin. Citral (a mixture of isomers with the *trans*-isomer geranial and *cis*-isomer neral), myrcene, and limonene are the major components of LEO [7]. These substances show biological activities including antioxidant, anti-inflammatory, and antibacterial properties [8,9]. Zulfa et al. (2016) [8] found that LEO exhibited different degrees of sensitivity in preventing the growth of several bacteria. Mounting evidence suggests that LEO has strong anticancer effects [10,11,12], making it an attractive research candidate.

In Thailand, lemongrass has long been used in Thai cuisine; however, its positive effects on several pathogenic bacteria and colon cancer are underexplored. Thus, this study investigated the antioxidant and antimicrobial activity of LEO from northeastern Thailand against four pathogens: *Escherichia coli* (food pathogen), *Cutibacterium acnes* (skin pathogen), *Streptococcus agalactiae* (fish pathogen), and *Staphylococcus aureus* (skin and food pathogen) and its anticancer capacity on HT-29 human colorectal adenocarcinoma cells. Our results will promote natural products as antioxidant, antimicrobial, and anticancer agents.

## 2. Materials and Methods

### 2.1. Plant Source and Extraction of Essential Oil

**Figure 1:**
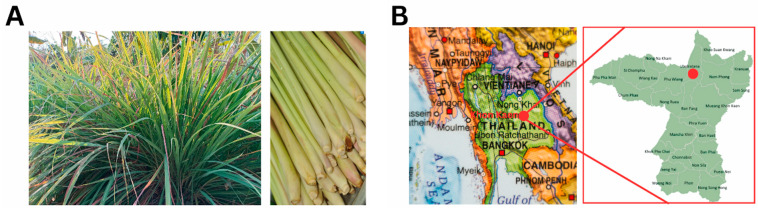
Lemongrass and collection location. (**A**) Leaves and *pseudostems* (**B**) Collection site at Ubolratana District (red dot), Khon Kaen Province (red font), Thailand.

Pseudostems and leaves of *C. citratus* were collected during the maturity season (Figure 1A) from local gardens under organic conditions in Ubolratana District, Khon Kaen Province, Thailand (Figure 1B) in September 2022 at 7.00 am. Taxonomy categorization of the lemongrass was conducted by Dr. Chadaporn Senakun, an assistant professor at Mahasarakham University’s Walai Rukhavej Botanical Research Institute. The voucher specimen BT092021 was submitted to the Natural Antioxidant Innovation Research Unit, Department of Biotechnology, Faculty of Technology, Mahasarakham University, Thailand. To extract LEO, 100 g of lemongrass pseudostems and leaves were washed, chopped into 1 cm-long pieces, and placed in a modified Clevenger-type steam system round-bottomed flask. Then, 300 mL of distilled water was added to the plant material, and LEO was obtained by hydrodistillation in a cyclic system for 4 h [13]. The hydrodistillation process yielded 0.81 g LEO from a 100 g lemongrass sample, giving a 0.81% yield of oil.

### 2.2. Gas Chromatography–Mass Spectrometry (GC-MS) 

Chemical analysis of LEO (1% *v*/*v*) dissolved in hexane was conducted using a Triple Quadrupole GC-MS/MS (GC-QQQ) Agilent Technologies, Agilent 7890B GC system, 7697A Static headspace sampler Agilent Technologies Headspace Model, Agilent 7633 ALS Autosampler and a fused silica HP5-MS capillary column (5% phenylmethylsiloxane, 30 m × 0.25 mm i.d.; film thickness, 0.25 μm) from Agilent, CA, USA, with a mass spectrometer as a detector. The injection volume was 0.5 µL, with a split ratio of 50:1, inlet temperature of 280 °C, helium carrier gas flow rate of 1.0 mL/min, solvent delay of 3 min, m/z scan range of 33–500, EI source temp. of 230 °C, and EI voltage of 70 eV. The oven was initially set at 80 °C for 2 min, then ramped up at 5 °C/min to 300 °C, held for 5 min (total 51 min) with a post-run at 310 °C (2 mL/min) for 5 min. Data were analyzed using MassHunter 2020 Acquisition. To positively identify the chemicals, the MS spectra were compared to NIST 2020 library reference spectra. The peak areas of all chemicals in the LEO were added to calculate their percentages.

### 2.3. Antioxidant Activity 

#### 2.3.1. Hydroxyl Radical Scavenging Capacity

A documented protocol was followed [14]. Fe^3+^-ascorbate-EDTA-H_2_O_2_, also known as the Fenton reaction, creates most hydroxyl radicals and measures 2-deoxy-D-ribose, a breakdown byproduct that appears pink when heated in an acidic environment with thiobarbituric acid (TBA). A phosphate buffer solution (50 mM, pH 7.4), LEO diluted with analytical-grade ethanol at concentrations of 20, 10, 5, 2.5, 1.25, 0.625, 0.3125, and 0.15625 mg/mL, EDTA (1.04 mM), FeCl_3_ (1 mM), and 2-deoxy-D-ribose (28 mM) comprised the components of the reaction mixture. The reaction was initiated by adding 0.2 mL of 2 mM ascorbic acid and 0.2 mL of 10 mM hydrogen peroxide to the mixture in a water bath at 37 °C. Following a 1 h incubation period, the reaction mixture was cooled with water before adding 1.5 mL of cold TBA (10 g/L) and 1.5 mL of 25% HCl. The mixture was heated at 100 °C for 15 min and cooled down. The solution absorbance was measured at 532 nm using a spectrophotometer. The inhibition of 2-deoxy-D-ribose oxidation was used to measure the antioxidant’s capacity to scavenge hydroxyl radicals. The IC_50_ value was calculated from a graph of hydroxyl radical scavenging capacity (%) against LEO concentrations, with the Trolox standard used as a positive control.

#### 2.3.2. DPPH Radical Scavenging Activity

A 100 µL aliquot of 0.2 mM DPPH methanolic solution was combined with 100 µL of LEO diluted with analytical-grade ethanol at concentrations of 20, 10, 5, 2.5, 1.25, 0.625, 0.3125, and 0.15625 mg/mL. After thorough mixing, the mixture was moved to a light-restricted environment for 15 min [15] and A_517_ nm was measured. The IC_50_ value was determined by plotting the percentage of radical scavenging activity against LEO concentration. The Trolox standard was used as a positive control.

### 2.4. Antimicrobial Activity 

Four pathogenic bacteria; *E. coli* ATCC 25922 (food pathogen; GenBank accession no. GCA_000401755.1), *C. acnes* DMST 14916 (skin pathogen; GenBank accession no. OQ368741.1), *S. agalactiae* EW1 (pathogen in fish; GenBank accession no. OR272051.1), and *S. aureus* TISTR 517 (skin and food pathogen; GenBank accession no. OP522324.1) were tested using the agar disk diffusion assay. Cultures of the four pathogenic bacteria were grown in Luria–Bertani (LB) broth (HiMedia, Maharashtra, India) for 24 h at 37 °C and set to 10^8^ CFU/mL using A_600_ nm. Paper discs (4 mm in diameter) were placed on LB agar plates with 100 µL inoculated culture. LEO (20 µL) at different concentrations was placed on the sample disks [16], which were then incubated at 37 °C for 24 h. The antimicrobial attribute was assessed by measuring the inhibition zone (mm in diameter). 

### 2.5. Cell Cultures 

HT-29 human colon cancer cells (ATCC^®^HTB-38, Manassas, VA, USA) were cultivated in Dulbecco’s Modified Eagle’s Medium (DMEM) supplemented with 10% fetal bovine serum (Gibco, Thermo Fisher Scientific, Inc., Carlsbad, CA, USA) as previously described [17].

### 2.6. Cytotoxicity Assay

The LEO was tested for cytotoxicity against an overnight growth of HT-29 cells (5 × 10^3^ cells/well) in 96-well plates using the MTT assay. The medium was replaced with MTT and allowed to respond for 4 h. The formazan crystals were dissolved in 200 µL DMSO and measured three times for absorbance at A_590_ nm using a microplate reader. Cell viability (%) and the IC_50_ values of HT-29 cells were assessed at 24 h and 48 h. Sigma-Aldrich, St. Louis, MO, USA supplied all the chemicals.

### 2.7. Colony Formation Assay

Five hundred HT-29 cells/well were cultured overnight. The cells were exposed to varying LEO concentrations in the medium for 24 h. After washing with PBS, the cells were cultured at 37 °C in 5% CO_2_ for 14 days. Freshly prepared medium was replaced daily. The cells were fixed for 30 min in −20 °C methanol. Coomassie brilliant blue g-250 was used to stain colonies for 30 min. The colony percentage of the treated cells was calculated, and the IC_50_ value was determined.

### 2.8. Cell Morphology

A total of 7500 HT-29 cells were seeded in each well of 24-well plates. Treatment of the cells with LEO (0–100 µg/mL) was initiated after 24 h. The cells were then immersed in cold methanol for cell fixation and stained with hematoxylin-eosin for 30 min before examination under a light microscope. 

### 2.9. Protein Isolation and Western Blot Experiment

The HT-29 cells were plated at 2.5 × 10^5^ cells/well in 6-well plates for 24 h before treatment with LEO at 50 μg/mL to examine their impact on protein expression using Western blot as previously described [17]. Live cells were washed with ice-cold PBS, lysed in RIPA buffer (50 mM Tris-Cl pH 7.4, 150 mM NaCl, 1% NP-40, 0.5% sodium deoxycholate, 0.1% SDS) with protease and phosphatase inhibitor cocktail (Roche, Penzberg, Germany) for 30 min on ice, and then centrifuged. After the supernatant was collected, a BCA protein kit test (Thermo Fisher Scientific, Rockford, IL, USA) was used to measure protein concentration. In a loading solution with 100 mM DTT, 20 µg of protein was boiled at 98 °C for 10 min. Protein samples were resolved by SDS-PAGE on 12% polyacrylamide gel electrophoresis and transferred to PVDF membrane at 90 V for 1 h. Rabbit polyclonal antibodies against human Bax, caspase-3, p21, and beta-actin were incubated overnight at 4 °C on membranes blocked for 1 h with 5% bovine serum albumin in Tris buffered saline (TBST). After three TBST washes, membranes were incubated at room temperature for 1 h with 1:5000 secondary Ab and horseradish peroxidase. After washing the membrane in TBST, Amersham ECL TM Prime was used to detect chemiluminescence. ChemiDoc Imaging Systems (Bio-Rad, Hercules, CA, USA) was used to monitor protein band densities. Triplicate was conducted.

### 2.10. Real-Time Polymerase Chain Reaction (RT-PCR)

Before LEO (25, 50, and 100 μg/mL) addition, HT-29 cells (2 × 10^5^/well) were cultured overnight. RT-PCR was used to examine mRNA expression after 24 h of testing the cancer cells. As previously described [16], total RNA was isolated using TRIzol^TM^ (Thermo Fisher Scientific, Waltham, MA, USA), with the cDNA produced and gene response evaluated by RT-PCR. 

### 2.11. Statistical Analysis

The means and standard deviations of triplicate data were determined using one-way ANOVA and Duncan’s multiple range test (SPSS, IBM, Armonk, NY, USA) using a significance level of *p* < 0.05.

## 3. Results

### 3.1. GC-MS Analysis of Bioactive Contents

**Figure 2:**
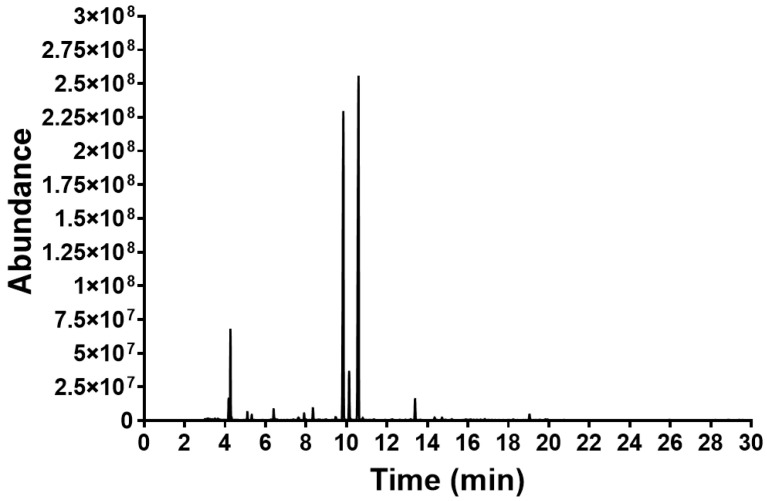
GC-MS chromatogram of LEO.

Forty compounds identified by GC-MS accounted for 98.79% of the abundance, with 19 peaks unidentified (1.21%) (Table 1). The LEO contained forty identifiable phytochemicals with seven prominent components (>1% abundance) identified as citral (79.24%) consisting of 44.52% geranial and 34.72% neral, β-myrcene (5.56%), geraniol (4.29%), geranyl acetate (1.82%), 6-methyl-5-hepten-2-one (1.36%), and 3,7-dimethyl-3,6-octadienal (1.09%) (Figure 2 and Table 1). Many substances have been shown to demonstrate anticancer action (Table 1). 

### 3.2. Antioxidant Activity

The LEO displayed low IC_50_ values for DPPH and hydroxyl scavenging assays, indicating strong antioxidant capacities (Table 2); however, the Trolox standard was stronger.

### 3.3. Antimicrobial Activity

**Figure 3:**
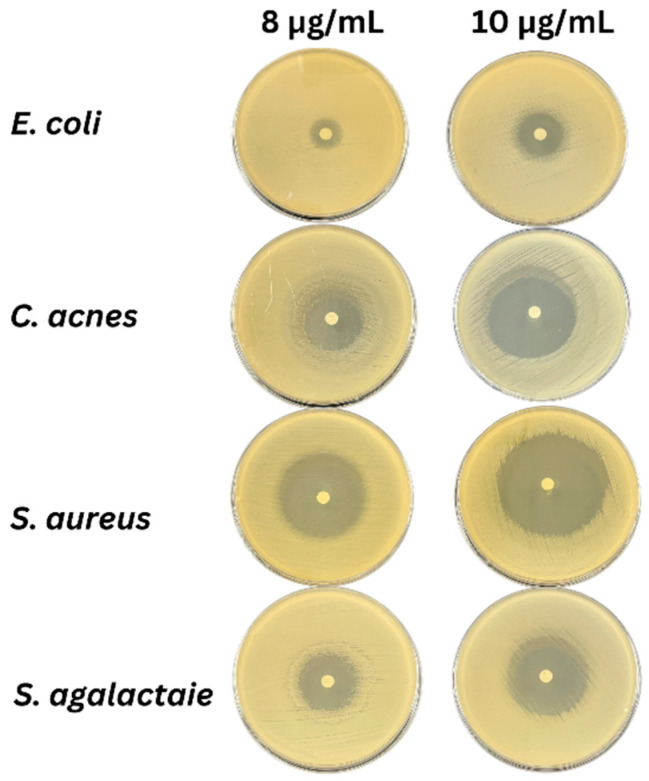
Inhibition zones (mm) of LEO against the four pathogenic bacteria.

The LEO was active against all four pathogenic bacteria (*E. coli*, *C. acnes*, *S. agalactiae*, and *S. aureus*) at 8 and 10 μg/mL (Table 3). The LEO showed thigher activity against Gram-positive bacteria, rather than the Gram-negative bacteria, with a larger clear zone of inhibition at 8 and 10 μg/mL doses as *S. aureus* > *C. Acnes* > *S. agalactiae* > *E. coli* (Figure 3).

### 3.4. Cytotoxicity of LEO toward HT-29 Cells

**Figure 4:**
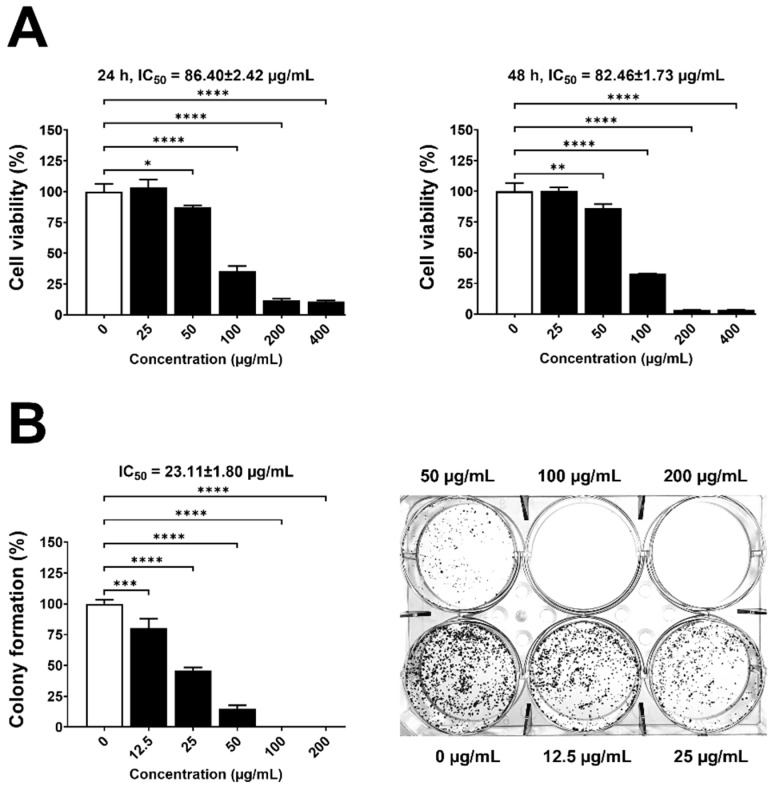
Effect of LEO on HT-29 cells. (**A**) Cell viability and (**B**) Colony formation. *, **, ***, and **** denote statistical significance at *p* < 0.05, <0.01, <0.001, and <0.0001.

The cytotoxicity of LEO toward HT-29 cells was dose-dependent and time-dependent. At 24 h, 12% cell viability was recorded at 200–400 µg/mL (IC_50_ value 86.40 ± 2.42 µg/mL) (Figure 4A, left panel). Low cell viability (3%) was observed at 48 h at 200–400 µg/mL (IC_50_ value 82.46 ± 1.73 µg/mL) (Figure 4A, right panel).

### 3.5. Antiproliferative Activity

The results of the dose-dependent clonogenic experiment demonstrated that LEO inhibited HT-29 cell proliferation after 14 days (Figure 4B). The clonogenic assay showed complete colony inhibition after treatment of 100 and 200 µg/mL LEO with a lower IC_50_ of 23.11 ± 1.80 µg/mL (Figure 4B). Results indicated that long-term LEO administration inhibited HT-29 cell proliferation.

### 3.6. Cell Morphological Changes

**Figure 5:**
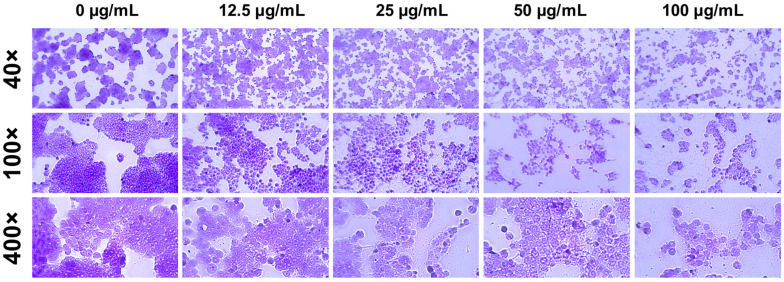
Effect of LEO on the morphological changes of HT-29 cells.

The density of HT-29 cells declined and apoptotic cell numbers and membrane blebbing increased in the treated cells at higher concentrations of LEO (Figure 5).

### 3.7. Effect of LEO on Apoptotic Genes and Protein Expressions

**Figure 6:**
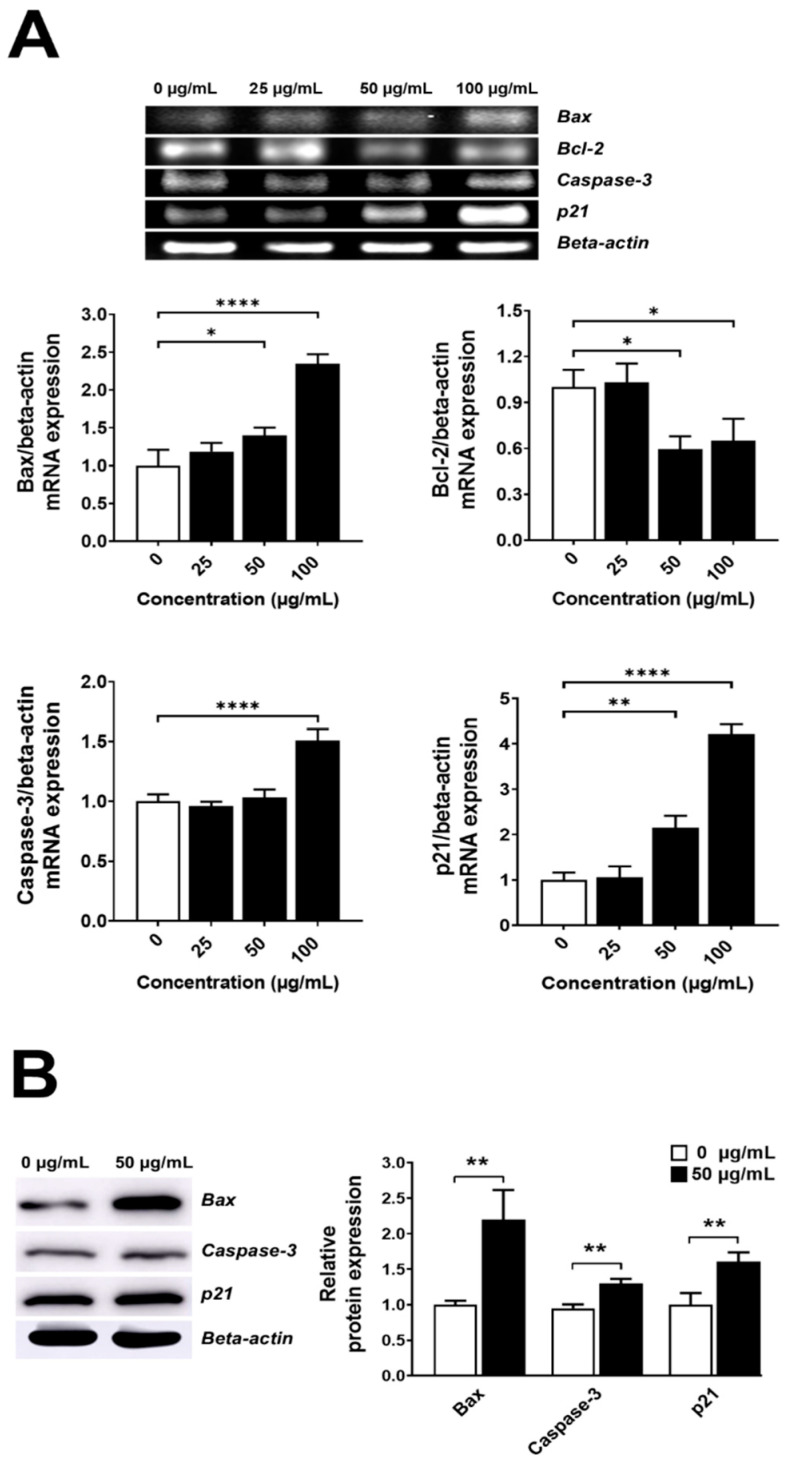
Effects of LEO on apoptosis-related gene and protein responses. (**A**) Genes, and (**B**) Proteins. *, **, ***, and **** denote statistical significance at *p* < 0.05, <0.01, <0.001, and <0.0001.

The LEO addition at 50 and 100 µg/mL caused significant decreases in the gene expression of *Bcl-2* while enhancing the expressions of *Bax*, *p21*, and *Caspase-3* (Figure 6A). The addition of LEO at 50 µg/mL substantially increased *Bax*, *p21*, and *Caspase-3* proteins (Figure 6B), indicating that LEO triggered intrinsic apoptosis.

## 4. Discussion

This study examined the chemical makeup of LEO extracted from plants grown in northeastern Thailand and assessed its antioxidant, antibacterial, and anticancer effects on HT-29 human colon cancer cells. The results revealed a wide range of chemicals in LEO, concurring with previous research. LEO mostly consists of citral, with concentrations ranging from 44.3% to 91.4% to 79% to 91.5% [7]. Other significant volatile components include β-myrcene (11%) and geraniol (1.9%) [40]. The GC-MS results identified citral (79.24%) consisting of 44.52% geranial and 34.72% neral, followed by β-myrcene (5.56%), geraniol (4.29%), geranyl acetate (1.82%), 6-methyl-5-hepten-2-one (1.36%), and 3,7-dimethyl-3,6-octadienal (1.09%) as the main components.

Antioxidant and antibacterial activities were shown by all of the primary ingredients [41], emphasizing the potential of LEO as an abundant reservoir of bioactive chemicals with medicinal uses. When compared with LEO from other countries, the antioxidant activity of LEO IC_50_ at 2.58 mg/mL from DPPH scavenging activity in this study was less effective than LEO from the north Indian plains, with an IC_50_ value of 0.5 mg/mL [42]. The chemicals in LEO differ depending on plant geographical source, extraction method, growth stage, and solvent used, thereby impacting its bioactivity [28].

Lemongrass extract showed varying levels of susceptibility in inhibiting the growth of *S. aureus*, *Bacillus cereus*, *Candida albicans*, and *E. coli* [8]. The antibacterial capabilities of the components in LEO differ depending on their functional groups. The most active components are phenols and aldehydes, while the least active components are esters and hydrocarbons [43]. In this study, LEO was effective against all four pathogens: *E. coli* (food pathogen), *C. acnes* (skin pathogen), *S. agalactiae* (fish pathogen), and *S. aureus* (skin and food pathogen) at 8 and 10 μg/mL due to the abundance of citral, β-myrcene, geraniol, geranyl acetate, and neo-intermedeol [37]. When comparing antimicrobial activity against *E. coli* with LEO from other countries, our LEO at 8 μg/mL was more effective than LEO from Malaysia, showing a minimum inhibitory concentration (MIC) of 0.63 mg/mL toward *E. coli* O157: H7 [8]. For *C. acnes* (formerly known as *Propionibacterium acnes*), our LEO at 8 μg/mL was more effective than LEO from Thai-China Flavors and Fragrances Industry Co. Thailand (Samut Prakan Province, Thailand), showing an MIC of 6.25 mg/mL [44]. Likewise, our LEO at 8 μg/mL was more effective against *S. agalactiae* than LEO from *Lavandula x intermedia* Emeric ex Loisel with MIC values of 9–18 μg/mL [45]. LEO from Heber Vietnam Co., Ltd. (Ho Chi Minh City, Vietnam) was effective against the *S. agalactiae* pathogen of red tilapia in Vietnam. Fish fed an LEO-supplemented diet at 200 mg LEO/kg demonstrated enhanced red blood cell production five days after *S. agalactiae* infection [46]. LEO in this study was also more effective against *S. aureus* than LEO from Vietnam, with an MIC of 5830 ± 198 µL/L [47]. The antibacterial properties of LEO indicated its usefulness as a natural substitute for synthetic antimicrobial agents, especially to counter increasing antimicrobial resistance.

The observed anticancer activity of LEO against HT-29 human colorectal adenocarcinoma cells is of particular relevance. Potential therapeutic uses of LEO in cancer management are highlighted by its significant decrease in viability and promotion of apoptosis. Phytochemicals in Table 1 responsible for the cytotoxic effects include geranial, neral, β-myrcene, geraniol, geranyl acetate, 6-methyl-5-hepten-2-one, and 3,7-dimethyl-3,6-octadienal. These induced apoptosis and inhibited cell proliferation among other anticancer mechanisms. In this study, the MTT assay showed LEO having an IC_50_ of 82.46 ± 1.73 µg/mL against HT-29 cells at 48 h. LEO from Thai-China Flavours and Fragrances Industry Co. Thailand showed decreased human colon cancer cell viability after 48 h (IC_50_ of 77.91 µg/mL) [48] which was more effective than our LEO. Premier Herbal Inc., of Toronto, ON, Canada, supplied a lemongrass extract (25–100 µg/mL) that promoted apoptosis in two colon cancer cells, HT-29 and HCT-116, in a time- and dose-dependent manner. Notably, this effect was observed without inducing damage to healthy cells in vitro. When administered orally to mice, lemongrass extract effectively inhibited the growth of colon cancer xenografts and was well tolerated [49]. Lemongrass extract exerted anticarcinogenic effects by inhibiting the proliferation of SW1417 human colon cancer cells (IC_50_ value of 150 μg/mL at 24 h) and inducing apoptosis through mitochondrial disruption and oxidative stress gene expression [50]. Compared with our results, LEO showed stronger cytotoxic activity against HT-29 colon cancer cells with an IC_50_ value of 82.46 ± 1.73 µg/mL at 48 h. This is the first report on LEO from northeastern Thailand showing cytotoxicity against HT-29 human colorectal adenocarcinoma cells using intrinsic apoptotic mechanisms. The clonogenic experiment demonstrated a reduced IC_50_ value of 23.11 µg/mL. Our results implied that long-term treatment with LEO could be used to prevent colon cancer relapse.

## 5. Conclusions

This study analyzed the contents of lemongrass *C. citratus* essential oil (LEO) from northeastern Thailand using GC-MS. Results revealed citral, β-myrcene, geraniol, geranyl acetate, 6-methyl-5-hepten-2-one, and 3,7-dimethyl-3,6-octadienal to be the primary phytochemicals. The LEO demonstrated antioxidant and antibacterial properties, with moderate cytotoxicity and substantial antiproliferative effects against HT-29 colon cancer cells. The stimulation of apoptosis mediated the cytotoxic impact of LEO. Our findings suggest that LEO has potential for various applications in the pharmaceutical, food, cosmetic, and health industries, focusing on colon cancer prevention and overall health benefits. The promising antiproliferative effects of LEO against HT-29 colon cancer cells warrant further investigation through preclinical studies and clinical trials. Additional investigations in this field are also necessary to maximize the therapeutic and commercial capabilities of LEO and its components.

## Figures and Tables

**Table 1:** GC-MS analysis of LEO chemical components.

Peak	RT	% Relative Abundance	Chemical’s Name	CAS No.	Bioactivity	Reference
1	3.709	0.02	Camphene; C_10_H_16_	79-92-5	Apoptosis in melanoma cells	[18]
2	4.173	1.36	6-Methyl-5-hepten-2-one; C_8_H_14_O	110-93-0	Anticancer	[19]
3	4.263	5.56	β-Myrcene; C_10_H_16_	123-35-3	Reduce colon inflammation	[20]
4	4.316	0.18	2,3-Dehydro-1,8-cineole; C_10_H_16_O	92760-25-3	Anti colon cancer	[21]
5	4.990	0.03	D-limonene; C_10_H_16_	5989-27-5	Anti colon cancer	[22]
6	5.058	0.02	Eucalyptol; C_10_H_18_O	470-82-6	Anti colon cancer	[21]
7	5.103	0.55	*cis*-3,7-Dimethyl-1,3,6-octatriene; C_10_H_16_	3338-55-4	Anti colon cancer	[23]
8	5.314	0.35	*trans*-β-Ocimene; C_10_H_16_	3779-61-1	Anti colon cancer	[24]
9	5.860	0.02	Linalool oxide; C_10_H_18_O_2_	5989-33-3	-	-
10	6.259	0.04	Unidentified	-	-	-
11	6.342	0.05	3-Methyl-2-(2-methyl-2-butenyl)-furan; C_10_H_14_O	15186-51-3	-	-
12	6.399	0.83	Linalool; C_10_H_18_O	78-70-6	Anti colon cancer	[25]
13	6.444	0.13	3-(4-Methyl-3-pentenyl)-furan; C_10_H_14_O	539-52-6	-	-
14	6.576	0.05	Unidentified	-	-	-
15	6.907	0.01	*trans*-1-Methyl-4-(1-methylvinyl)cyclohex-2-en-1-ol; C_10_H_16_O	7212-40-0	-	-
16	7.076	0.04	2,6-Dimethyl-2,4,6-octatriene; C_10_H_16_	673-84-7	-	-
17	7.351	0.05	Unidentified	-	-	-
18	7.442	0.04	7-Methyl-3-methyleneoct-6-enal; C_10_H_16_O	55050-40-3	-	-
19	7.596	0.11	*trans*-Chrysanthemal; C_10_H_16_O	20104-05-6	Anti colon cancer	[26]
20	7.634	0.24	Citronellal; C_10_H_18_O	106-23-0	Anti colon cancer	[27]
21	7.905	0.53	Isoneral; C_10_H_16_O	1000414-18-0	Anticancer	[28]
22	7.995	0.06	Unidentified	-	-	-
23	8.172	0.03	2-((3,3-Dimethyloxiran-2-yl)methyl)-3-methylfuran; C_10_H_14_O_2_	92356-06-4	-	-
24	8.342	1.09	3,7-Dimethyl-3,6-octadienal; C_10_H_16_O	55722-59-3	-	-
25	8.598	0.05	Unidentified	-	-	-
26	8.666	0.07	Unidentified	-	-	-
27	8.835	0.03	(-)-*trans*-Isopiperitenol; C_10_H_16_O	74410-00-7	-	-
28	8.982	0.08	Carveol; C_10_H_16_O	99-48-9	Anti breast cancer	[29]
29	9.284	0.04	(-)-*cis*-Isopiperitenol; C_10_H_16_O	96555-02-1	-	-
30	9.464	0.35	Citronellol; C_10_H_20_O	106-22-9	Anti colon cancer	[27]
31	9.841	34.72	Neral; C_10_H_16_O	106-26-3	Anti colon cancer	[30]
32	10.127	4.29	Geraniol; C_10_H_18_O	106-24-1	Anti colon cancer	[31]
33	10.594	44.52	Geranial; C_10_H_16_O	141-27-5	Anti colon cancer	[32]
34	10.802	0.17	Unidentified	-	-	-
35	11.351	0.06	Geranyl formate; C_11_H_18_O_2_	105-86-2	Anti colon cancer	[33]
36	12.255	0.1	Unidentified	-	-	-
37	12.624	0.04	Unidentified	-	-	-
38	12.911	0.04	Unidentified	-	-	-
39	13.163	0.11	Unidentified	-	-	-
40	13.385	1.82	Geranyl acetate; C_12_H_20_O_2_	105-87-3	Anti colon cancer	[33]
41	13.66	0.05	Unidentified		-	-
42	14.357	0.31	Caryophyllene; C_15_H_24_	87-44-5	Anti cancer	[34]
43	14.719	0.24	2,6-Dimethyl-6-(4-methyl-3-pentenyl)bicyclo [3.1.1]hept-2-ene; C_15_H_24_	17699-05-7	-	-
44	14.9	0.04	Unidentified	-	-	-
45	15.19	0.05	Humulene; C_15_H_24_	6753-98-6	Anti colon cancer	[35]
46	15.905	0.06	4a,8-Dimethyl-2-(prop-1-en-2-yl)-1,2,3,4,4a,5,6,7-octahydronaphthalene; C_15_H_24_	103827-22-1	-	-
47	16.078	0.03	2,3,4,4a,5,6-Hexahydro-1,4a-dimethyl-7-(1-methylethyl) naphthalene; C_15_H_24_	473-14-3	-	-
48	16.131	0.08	Unidentified	-	-	-
49	16.286	0.04	Unidentified	-	-	-
50	16.433	0.06	Unidentified	-	-	-
51	16.625	0.05	1-Isopropyl-7-methyl-4-methylene-1,2,3,4,4a,5,6,8a-octahydronaphthalene;C_15_H_24_	39029-41-9	-	-
52	16.832	0.09	1,2,3,5,6,8a-Hexahydro-4,7-dimethyl-1-(1-methylethyl)naphthalene; C_15_H_24_	483-76-1	-	-
53	18.241	0.09	Unidentified	-	-	-
54	19.041	0.62	Selin-6-en-4α-ol; C_15_H_26_O	118173-08-3	-	-
55	19.54	0.05	Unidentified	-	-	-
56	19.834	0.08	α-Cadinol; C_15_H_26_O	481-34-5	Anti colon cancer	[36]
57	19.939	0.08	Neointermedeol; C_15_H_26_O	5945-72-2	Antibacterial Gram + bacteria	[37]
58	20.749	0.03	1,4a-Dimethyl-7-(1-methylethylidene)decahydro-1-naphthalenol; C_15_H_26_O	473-04-1	Anti colon cancer	[38]
59	25.966	0.04	m-Camphorene; C_20_H_32_	20016-73-3	Anticancer	[39]
60	26.629	0.02	Unidentified	-	-	-

RT = retention time.

**Table 2:** IC_50_ values of LEO antioxidant activities.

Sample	IC_50_ (mg/mL)	
	DPPH Scavenging Activity	Hydroxyl Scavenging Activity
LEO	2.58 ± 0.08 ^b^	4.05 ± 0.12 ^b^
Trolox	0.17 ± 0.00 ^a^	0.08 ± 0.00 ^a^

Different superscripts ^a, b^ in the same column between LEO and the Trolox standard indicate statistical significance (*p* < 0.05).

**Table 3:** Antimicrobial activity of LEO.

Pathogenic Bacteria		Inhibition Zone (mm) of LEO				
		2 μg/mL	4 μg/mL	6 μg/mL	8 μg/mL	10 μg/mL
Gram −	*E. coli*	nd	nd	nd	13.00 ± 1.00 ^Bd^	23.33 ± 0.58 ^Ad^
Gram +	*C. acnes*	nd	nd	nd	24.33 ± 2.52 ^Bb^	42.33 ± 4.04 ^Ab^
	*S. aureus*	nd	nd	nd	45.00 ± 0.00 ^Ba^	50.67 ± 4.04 ^Aa^
	*S. agalactiae*	nd	nd	nd	16.67 ± 4.16 ^Bc^	29.33 ± 0.58 ^Ac^

Different capital superscripts ^A, B^ in the same row among different doses and different small superscripts ^a, b, c, d^ in the same column among different pathogenic bacteria at the same dosage indicate statistical significance (*p* < 0.05). nd = not detected.

## Data Availability

The raw data supporting the conclusions of this article will be made available by the authors on request.
